# Exploring the Feasibility of Fall Detection Using Bluetooth Low Energy Channel Sounding in Residential Environments

**DOI:** 10.3390/s26061930

**Published:** 2026-03-19

**Authors:** Šarūnas Paulikas, Simona Paulikiene

**Affiliations:** 1 Department of Computer Science and Communication Technologies, Faculty of Electronics, Vilnius Gediminas Technical University, LT-10223 Vilnius, Lithuania; 2 Nursing Department, Faculty of Health Care, Vilniaus kolegija—Higher Education Institution, LT-08105 Vilnius, Lithuania

**Keywords:** fall detection, Bluetooth Low Energy, channel sounding, device-free sensing, ambient assisted living, radio-frequency sensing, privacy-preserving monitoring, machine learning, sensitivity-first evaluation

## Abstract

Falls represent a major health risk for older adults living independently, motivating the development of unobtrusive and privacy-preserving monitoring solutions. This study investigates whether Bluetooth Low Energy (BLE) 6.0 Channel Sounding (CS) can support device-free fall detection using low-complexity signal representations suitable for residential deployment. The proposed system employs two BLE nodes performing periodic channel sounding, from which only scalar distance estimates are extracted. Time-domain and temporal-dynamic features are computed from sliding windows of the distance signal and used for supervised classification. Three widely used classifiers—Support Vector Machine with radial basis function kernel, Random Forest, and gradient-boosted decision trees (XGBoost)—are evaluated under both a default operating point and a sensitivity-first regime achieved through validation-based decision threshold adjustment, reflecting the higher cost of missed fall detections in assisted living scenarios. Experiments conducted in a furnished indoor environment with six participants performing realistic fall and non-fall scenarios demonstrate strong window-level sensitivity under subject-independent evaluation, with XGBoost providing the most favorable sensitivity–specificity balance. Under sensitivity-first operation, very high recall is achieved at the expense of increased false alarms. Given the limited dataset and single-environment setting, the reported results should be interpreted as a proof-of-concept demonstration of feasibility rather than definitive large-scale performance. The findings suggest that BLE CS captures motion-relevant signal variations that may support practical fall detection while maintaining low deployment complexity and privacy preservation.

## 1. Introduction

Population ageing is one of the most significant demographic challenges facing modern societies. In Europe, the proportion of individuals aged 65 years and older continues to increase, while a growing share of this population lives alone due to changing family structures, migration, and urbanization trends [1,2]. This development places substantial pressure on healthcare and social care systems and increases the vulnerability of elderly individuals to adverse events in domestic environments. Among these events, falls represent one of the most frequent and severe risks, often leading to serious injuries, long-term disability, loss of independence, or death [3]. Importantly, the consequences of falls are strongly influenced by the time required to provide assistance, a factor that is particularly critical for elderly people living alone [4].

Fall detection systems have therefore become a central research topic within the domains of Ambient Assisted Living (AAL) and smart home technologies [5]. The primary objective of these systems is to automatically detect fall events and trigger timely alerts to caregivers, relatives, or emergency services, thereby reducing medical complications and improving quality of life. Early research and many commercial solutions have relied predominantly on wearable sensors, such as accelerometers and gyroscopes embedded in pendants, wristbands, or smartphones [6]. Although wearable-based approaches can achieve high detection accuracy under controlled conditions, their real-world applicability is limited by several factors, including low user compliance, discomfort, battery maintenance, and the tendency of elderly users to forget or refuse wearing the devices [7].

An alternative class of solutions employs vision- and audio-based sensing, including RGB cameras, depth sensors, and microphones. These systems can provide rich contextual information and high recognition performance, particularly when combined with machine learning and deep learning techniques [8]. However, their deployment in private homes raises significant privacy, ethical, and legal concerns, especially in sensitive areas such as bedrooms and bathrooms. Within the European Union, such concerns are reinforced by the General Data Protection Regulation (GDPR), which requires strict data minimization, purpose limitation, and privacy-by-design principles when processing personal data [9]. Continuous visual or acoustic monitoring is often perceived as intrusive and has been shown to negatively affect user acceptance, creating a major barrier to large-scale adoption of camera- or microphone-based fall detection systems [10].

These limitations have motivated increasing interest in device-free and privacy-preserving fall detection approaches that avoid wearable devices and do not rely on cameras or microphones. In recent years, wireless sensing techniques based on radio-frequency (RF) signals have emerged as a promising alternative. Technologies such as Wi-Fi channel state information (CSI), Bluetooth Low Energy (BLE), ultra-wideband (UWB), and millimeter-wave radar enable the detection of human motion and posture changes by analyzing variations in electromagnetic wave propagation caused by the human body [11,12]. Because these approaches do not capture identifiable visual or acoustic information, they are inherently more compatible with EU privacy regulations and social expectations regarding in-home monitoring [13].

Among RF-based approaches, Wi-Fi and Bluetooth are particularly attractive due to their widespread availability in residential environments, low deployment cost, and minimal user interaction requirements. Wi-Fi-based sensing has demonstrated strong potential for device-free fall detection by exploiting channel state information to capture motion-induced variations in signal amplitude and phase [11,14]. In contrast, traditional Bluetooth-based sensing relying on received signal strength indicator (RSSI) measurements typically provides coarser spatial resolution and limited sensitivity to fine-grained motion dynamics. Nevertheless, Bluetooth offers complementary advantages such as low power consumption, flexible node placement, and ease of deployment, making it well suited for long-term residential monitoring.

Recent studies indicate that combining multiple sensing modalities can improve robustness and reliability in complex indoor environments [12,15]. At the same time, increasing system complexity through additional sensors or specialized hardware may conflict with the practical requirement for low-cost, easy-to-install solutions suitable for elderly users and non-technical caregivers.

Motivated by recent advances in device-free RF sensing and the introduction of CS in BLE version 6.0, this work investigates the feasibility of using Bluetooth-based sensing for fall detection in residential environments. BLE CS enables standardized phase- and time-based radio channel measurements using low-power devices, offering sensing capabilities conceptually closer to Wi-Fi CSI while retaining the deployment advantages of Bluetooth technology. The proposed approach aims to achieve privacy preservation, low energy consumption, and minimal installation complexity, while prioritizing fall detection sensitivity over false alarm reduction.

The remainder of this paper is organized as follows. Section 2 reviews related work on fall detection, with emphasis on privacy-preserving and device-free sensing approaches. Section 3 introduces the principles of BLE CS and outlines the proposed sensing framework. Section 4 describes the experimental methodology, including the hardware setup, data collection protocol, feature extraction, classification models, and evaluation metrics. Experimental results are presented and analyzed in Section 5, followed by a discussion of limitations and practical implications in Section 6. Finally, Section 7 concludes the paper and outlines directions for future work.

## 2. Related Work

Research on fall detection in Ambient Assisted Living and smart home environments has explored a wide range of sensing paradigms, including wearable sensors, vision-based systems, device-free wireless sensing, and infrastructure-integrated solutions. In addition to commonly studied approaches, systems based on smart floors, pressure-sensitive carpets, and vibration-aware flooring have also been proposed [16,17]. These solutions can achieve reliable detection by directly sensing ground reaction forces or vibration patterns caused by falls. However, their deployment typically requires significant structural modifications, specialized materials, and professional installation, which substantially increases cost and limits scalability. Consequently, such approaches are generally considered impractical for widespread deployment in existing residential buildings, particularly private homes and rental apartments [5].

Wearable-based fall detection systems predominantly rely on inertial measurement units (IMUs), such as accelerometers and gyroscopes, embedded in smartphones, smartwatches, or dedicated pendants [6]. These approaches benefit from direct measurement of body dynamics and have been extensively studied over the past two decades. Although high detection accuracy is often reported under controlled conditions, real-world performance is strongly affected by user compliance, correct sensor placement, battery maintenance, and long-term comfort [7]. These limitations are particularly relevant for elderly individuals with cognitive decline, who may forget or refuse to wear the devices. Recent systematic reviews indicate that, on average, wearable-based solutions exhibit lower sensitivity and robustness than non-wearable and hybrid approaches when evaluated across diverse scenarios [5]. Furthermore, many wearable-based methods are trained and tested using datasets consisting primarily of simulated falls performed by young or healthy subjects, which limits generalization to frail older adults and realistic domestic environments [18].

Vision-based fall detection systems employ RGB cameras, depth sensors, thermal cameras, or multi-camera configurations to recognize posture changes and fall events [8]. Advances in deep learning for human pose estimation and action recognition have enabled high classification accuracy in experimental settings. However, the applicability of vision-based solutions in private homes is constrained by privacy, ethical, and legal concerns, particularly in sensitive areas such as bedrooms and bathrooms [10]. Within the European Union, these concerns are reinforced by the General Data Protection Regulation (GDPR), which mandates strict data minimization and privacy-by-design principles [9]. From a technical perspective, vision-based systems are sensitive to illumination changes, occlusions, camera placement, and field-of-view limitations. Although depth and thermal imaging reduce identifiability compared to RGB video, they remain environment-dependent and do not fully resolve privacy concerns [8].

To address privacy and usability limitations, device-free wireless sensing approaches based on radio-frequency (RF) signals have gained increasing attention. Wi-Fi-based fall detection systems exploit variations in channel state information (CSI) caused by human motion to infer activities without requiring users to wear devices or be visually monitored [19]. Numerous studies demonstrate the feasibility of CSI-based fall detection using signal preprocessing, time–frequency analysis, and machine learning or deep learning classifiers [14]. However, a key challenge remains environment dependence: models trained in one home often experience performance degradation when furniture layouts, device placement, or propagation conditions change. The DeFall system explicitly addresses this issue by proposing environment-independent features derived from motion dynamics and reports improved robustness compared to earlier CSI-based approaches [11]. Recent analyses further indicate that performance reported on limited or overly controlled datasets may substantially overestimate real-world accuracy in WiFi-based human activity recognition [20].

Bluetooth-based sensing has traditionally been limited to coarse measurements such as the received signal strength indicator (RSSI), which significantly constrains its applicability for fine-grained human activity recognition and fall detection due to low temporal resolution and strong sensitivity to environmental variability [21]. In contrast to Wi-Fi CSI, legacy BLE metrics lack access to stable phase- and subcarrier-level information required for detailed motion characterization.

Recent advances in the Bluetooth Core Specification, particularly the introduction of CS in BLE version 6.0, enable synchronized phase- and time-based channel measurements that are conceptually closer to Wi-Fi CSI than legacy BLE metrics [22,23]. To date, BLE CS has been primarily investigated for high-accuracy ranging, positioning, and localization applications [23,24], while its potential for device-free human activity recognition and fall detection remains largely unexplored. This gap motivates the investigation of BLE CS as a future-compatible sensing modality for privacy-preserving fall detection systems that combine low deployment cost, energy efficiency, and improved sensing fidelity compared to RSSI-based approaches.

Millimeter-wave and FMCW radar-based fall detection systems represent another class of device-free, privacy-preserving approaches. These systems can extract range, Doppler, and micro-motion features that are robust to lighting conditions and have demonstrated high detection accuracy under controlled conditions [25]. Multi-view radar configurations further improve robustness with respect to viewing angle and subject orientation. Nevertheless, radar-based solutions typically require dedicated hardware, careful calibration, and higher deployment cost compared to commodity wireless technologies such as Wi-Fi or Bluetooth, which limits their practicality for low-cost residential installations.

Hybrid and multimodal systems combine multiple sensing modalities to improve detection reliability and reduce false alarms. Recent reviews report that hybrid solutions often achieve the highest overall sensitivity and robustness among fall detection approaches [5]. However, these gains are achieved at the expense of increased system complexity, synchronization overhead, installation effort, and maintenance requirements, which may conflict with the goal of developing low-cost, easy-to-install solutions suitable for elderly individuals living independently [15].

This work is positioned to address several persistent shortcomings identified in the literature. First, many existing methods are validated primarily on simulated falls performed by young volunteers in laboratory settings. To improve ecological validity, we train and evaluate models using realistic fall scenarios performed by trained actors, with fall mechanisms informed by interviews with paramedics who routinely respond to real fall incidents. Second, although multi-person activity recognition is an important research topic, it is not the primary focus of this study. The proposed system targets elderly individuals living alone, for whom fall detection is most critical. Finally, this work investigates a device-free approach based on emerging BLE CS, leveraging standardized phase-based measurements to enable privacy-preserving fall detection without requiring cameras, microphones, or wearable devices.

Building on these insights, the following section introduces the proposed sensing methodology and experimental design.

## 3. Bluetooth Channel Sounding for Device-Free Sensing

BLE CS introduces a standardized mechanism for phase-based ranging by exchanging unmodulated tones between two BLE devices operating in Initiator and Reflector roles. During a CS procedure, phase measurements are obtained across multiple frequency channels. Under line-of-sight (LOS) conditions, the phase response exhibits an approximately linear progression with respect to frequency, where the slope is proportional to the signal propagation delay. By estimating this delay and converting it using the speed of light, a distance estimate between the devices is derived.

In static environments, the measured phase slope and the derived distance estimate remain stable over time. However, when a person or object moves within the propagation environment, multipath components are altered due to reflection, scattering, and shadowing. These changes introduce time-varying perturbations in the measured phase and distance estimates, even though the physical locations of the devices remain fixed.

Phase-based ranging is inherently sensitive to multipath propagation and non-line-of-sight (NLOS) conditions. In NLOS scenarios, excess path length caused by reflections may introduce positive bias in the estimated distance. Antenna orientation and polarization mismatch can also influence the effective channel response and modify the phase characteristics across frequency. In dynamic human scenarios, body movement continuously reshapes the multipath structure, resulting in transient fluctuations of the estimated distance. Rather than treating these fluctuations purely as noise, device-free sensing approaches intentionally exploit such motion-induced channel perturbations as informative temporal features.

BLE CS supports several ranging approaches, including received signal strength (RSS)-based estimation, phase-based ranging (PBR), and time-of-flight (ToF) techniques. The achievable ranging accuracy and stability depend on both the signal processing method and the environmental propagation conditions. Recent studies have analyzed the metrological properties of these approaches and the design trade-offs between them [26]. In particular, PBR-based methods exploit phase differences across multiple frequencies to estimate propagation distance and are considered one of the most promising techniques for short-range indoor localization.

Several recent works have investigated improvements to BLE CS distance estimation through advanced signal processing and machine learning approaches [27,28,29]. Experimental evaluations using commercial hardware also show that the achievable performance depends strongly on implementation details and environmental conditions, including multipath propagation and interference in the 2.4 GHz band [30,31].

Recent studies have shown that such perturbations can be exploited for device-free sensing, including presence detection and activity recognition, without requiring the target to carry any wireless device. In particular, the work in [23] demonstrates that CS phase and amplitude variations can serve as sensing signals analogous to Wi-Fi CSI, while offering improved energy efficiency and native support in Bluetooth 6.0-compliant devices.

In the present study, the goal is not to develop a new ranging algorithm but to explore the feasibility of using BLE CS measurements for device-free fall detection. The experiments rely on the scalar distance estimate produced by the controller implementation of phase-based ranging on nRF54L15 development boards from Nordic Semiconductor ASA, Trondheim, Norway. The devices operate using the BLEs CS procedure implemented in the nRF Connect SDK, where one device acts as the Initiator and the other as the Reflector. During each CS procedure, the Initiator transmits sounding packets across multiple frequencies in the 2.4 GHz band and measures the resulting phase responses exchanged between the two devices. These phase measurements are processed internally by the controller firmware to estimate the propagation distance between the devices.

The CS procedure is periodically executed according to configuration parameters provided by the Nordic SDK, which define the measurement interval and the number of channel sounding exchanges. After each completed CS procedure, the Initiator reports a single distance estimate to the host application. In the experimental setup, this value is logged together with a timestamp and stored as a time series representing the estimated separation between the two devices.

Raw channel measurements, such as individual phase samples, per-tone measurements, or in-phase and quadrature (I/Q) data, are not exposed by the controller interface and were therefore not recorded. Consequently, the signal processing and machine learning stages operate exclusively on the controller-provided distance estimates. The fall detection approach proposed in this work analyzes the temporal variations of this distance signal, which are introduced by human motion within the sensing region between the Initiator and Reflector devices.

In this work, CS is employed as a privacy-preserving sensing modality for fall detection. The controller-provided distance estimates are treated as a compressed representation of the underlying multipath dynamics and analyzed over time to detect motion patterns associated with falls. This design choice enables lightweight processing while maintaining compatibility with commercially available Bluetooth 6.0 hardware.

## 4. Design of Fall Scenarios and Experimental Setup

The experimental design aims to evaluate the feasibility of detecting falls using BLE CS in a device-free and privacy-preserving manner. The experiments are structured to capture realistic fall dynamics, include challenging non-fall activities that may lead to false alarms, and reflect typical residential environments for older adults living alone.

### 4.1. Experimental Environment and Hardware Setup

All experiments were conducted in indoor residential-like environments characterized by rich multipath propagation, including typical household furniture and partial non-line-of-sight (NLOS) conditions. The experiments were designed to closely resemble realistic home deployments, rather than controlled laboratory settings, in order to capture signal dynamics representative of real-world operation.

The sensing system consists of two BLE devices implementing BLE CS:

- An *Initiator* node connected via USB to a host computer for control, data logging, and offline analysis;
- A *Reflector* node placed at a fixed location within the environment and powered continuously.

Both nodes are implemented using Nordic Semiconductor nRF54L15 development boards, which natively support BLE CS and are compatible with the nRF Connect SDK (version 3.0.1 and later). The Initiator periodically executes CS procedures, during which phase-based ranging is performed using synchronized frequency and timing exchanges between the Initiator and Reflector. For the baseline experiments reported in this work, only the resulting distance estimates are logged, while raw in-phase and quadrature (I/Q) samples or per-tone phase-based ranging (distance estimation) are not stored.

The Initiator is connected to a host computer (Apple MacBook equipped with an M-series processor) via USB, which provides both power and a reliable high-throughput interface for command execution and data acquisition. All experiment control, time-stamping, and data storage are performed on the host computer, enabling reproducible measurements and consistent data management.

Both BLE devices are mounted at an approximate height of 1.2 m above the floor, corresponding to typical wall-mounted or shelf-mounted installation in residential settings. The horizontal separation between the Initiator and Reflector is approximately 6 m, reflecting practical constraints of medium-sized rooms or adjacent-room deployments. This configuration ensures that the measured CS distance estimates are influenced by human motion within the Fresnel zone between the devices, while remaining feasible for unobtrusive installation in real homes.

Ground-truth activity labels were obtained using synchronized video recordings, as described in Section 4.5.

### 4.2. Apartment Environment and Living Room Setup

**Figure 1:**
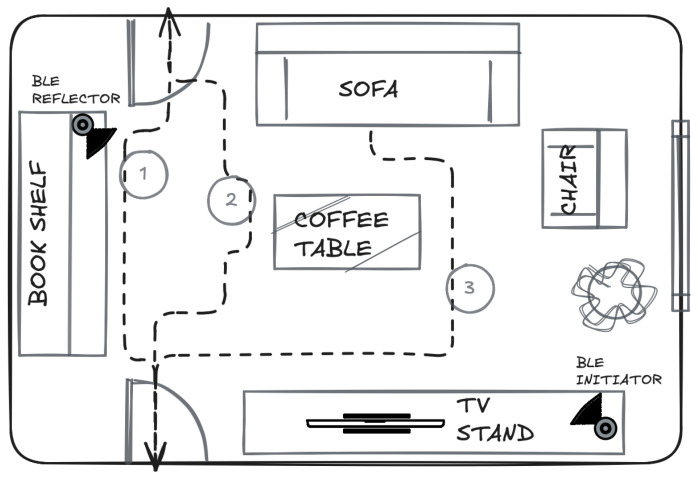
Representative living room layout (approx. 24 m^2^) used for scenario-based data collection. The setup includes a sofa, coffee table, bookshelf or storage unit, and TV stand or low cabinet, enabling wall- and furniture-supported balance recovery attempts and realistic fall trajectories. Dashed paths illustrate the typical movement trajectories of the three fall and non-fall scenarios evaluated in this study.

Data collection was conducted in a representative apartment living room environment rather than in an empty laboratory space. The room layout and furniture arrangement are illustrated in Figure 1: the room is approx. 6 and 4 m, with an area approximately 24 m^2^, corresponding to typical living spaces occupied by single older adults.

The furnished environment includes:

- a sofa used for sit-to-stand transitions,
- a coffee table,
- a bookshelf or storage unit,
- a TV stand or low cabinet,
- optional elements such as a chair or plant.

Furniture was arranged to create realistic movement paths, partial line-of-sight obstructions, and natural support points. This setup ensures that interactions with the environment generate realistic motion patterns and sensing conditions, including multipath effects relevant for radio-frequency-based sensing approaches.

### 4.3. Dataset Size Considerations and Prior Work Context

Existing experimental datasets for non-wearable fall detection are often limited in size and diversity, which may negatively affect generalization to real-world deployments. For example, a deep-learning-based Wi-Fi CSI study reported a dataset of over 700 CSI samples collected across four indoor environments and multiple fall and daily activity types [14]. Environment-independent Wi-Fi fall detection has also been evaluated using extensive dummy-based experiments comprising several hundred fall trials, supplemented by additional experiments involving real human falls [11].

Beyond radio-frequency sensing, privacy-aware multimodal approaches combining thermal cameras and wearable sensors have reported sequence-level datasets with tens of fall and non-fall sequences recorded from multiple viewpoints [10]. Recent studies have further shown that performance reported on limited or overly controlled datasets may substantially overestimate real-world accuracy in Wi-Fi-based human activity recognition [20].

These observations motivate the present work to emphasize (i) realistic home interactions involving walls, furniture, and transitional movements, and (ii) challenging non-fall activities that closely resemble fall dynamics, rather than relying solely on simplified or highly controlled experimental setups.

Although the resulting dataset is modest in absolute size, it is comparable to or larger than several prior device-free fall detection studies that emphasize realistic environments over large-scale data collection. Unlike vision-based approaches that can generate thousands of frames per trial, BLE CS produces compact, low-dimensional time-series data, where each trial yields a limited number of statistically meaningful windows. In this context, increasing dataset size by simply repeating highly similar trials provides diminishing returns and may artificially inflate performance.

Instead, this work prioritizes diversity of motion patterns, environmental interactions, and subject-independent evaluation. By including multiple fall mechanisms, paired non-fall activities, realistic furniture interactions, and unseen-subject testing, the dataset is designed to stress generalization rather than maximize sample count. This design choice aligns with recent findings that performance reported on large but homogeneous RF sensing datasets may substantially overestimate real-world accuracy when deployed in unconstrained home environments [20].

### 4.4. Fall Scenario Design

Most existing fall detection studies rely on simplified and highly controlled laboratory scenarios, where falls are performed in empty rooms with minimal interaction with the surrounding environment. In contrast, real-world falls in domestic settings rarely occur under such conditions. Older adults frequently attempt to recover balance by leaning on walls, grabbing furniture, or using assistive objects, and loss of balance often occurs during transitional movements such as standing up or reaching. These interactions substantially influence motion dynamics and sensing signatures, yet they remain insufficiently represented in many publicly available fall detection datasets.

To address these limitations, this work adopts a scenario design strategy focused on realistic fall and non-fall events performed in furnished living spaces. Each fall scenario is explicitly paired with one or more kinematically and contextually similar non-fall activities, encouraging the learning of discriminative patterns rather than relying on exaggerated or isolated motion signatures. All scenarios were performed by actors, and the selected fall mechanisms were informed by real-life cases collected through interviews with paramedics routinely responding to fall-related emergency calls.

**Scenario 1: Walking with wall or shelf interaction.** In the fall case, the subject walks forward, loses balance, makes contact with a shelf using the hand or shoulder, and subsequently slides to the floor. In the corresponding non-fall case, the subject walks while intentionally leaning on a shelf or wall for support, maintains balance, and continues movement. This scenario reflects common situations near room boundaries, where wall contact alone does not necessarily indicate a fall.

**Scenario 2: Walking with furniture support.** In the fall case, the subject walks, stumbles, attempts to stabilize by grabbing a piece of furniture (e.g., a table, shelf, or walker), fails to recover balance, and collapses to the floor. In the non-fall case, the subject walks while actively using furniture as support without losing balance. Furniture-assisted locomotion is frequent among older adults, and unsuccessful recovery attempts are a key characteristic distinguishing falls from normal supported walking.

**Scenario 3: Sit-to-stand transition from a sofa.** In the fall case, the subject attempts to stand up from a sofa, loses balance during the transition, and collapses or slides to the floor. In the non-fall case, the subject successfully stands up from the sofa using normal or assisted motion and continues walking. Sit-to-stand transitions are among the most critical movements associated with fall risk and are often underrepresented in fall detection datasets.

### 4.5. Static Baseline Characterization

To quantify the intrinsic variability of the phase-derived distance estimate in the absence of human motion, a static baseline measurement was conducted in the same environment with no test subjects present. CS measurements were recorded over a duration comparable to that of typical experimental trials while the devices remained fixed and the room unoccupied.

Under static conditions, the estimated distance exhibited a mean value of 5.940 m with a standard deviation of 0.058 m (approximately 5.8 cm). This short-term variability reflects residual multipath sensitivity, oscillator phase noise, and internal estimation uncertainty inherent to phase-based ranging.

Occasional larger excursions were observed over extended recording durations, consistent with slow multipath fluctuations typical of indoor radio environments. Nevertheless, the standard deviation establishes an intrinsic measurement noise floor for the sensing system under no-motion conditions.

In comparison, fall events produced transient deviations in the distance signal substantially exceeding this baseline standard deviation. The magnitude and temporal dynamics of these deviations were therefore significantly above the intrinsic measurement variability, supporting the use of distance-based temporal features for fall detection.

It is important to emphasize that the distance signal used in this study is obtained directly from the controller implementation of phase-based ranging in the BLE CS stack rather than from a custom ranging algorithm developed by the authors. The Nordic nRF54L15 platform internally processes phase measurements exchanged during CS procedures and reports a scalar distance estimate to the host application. As a result, the absolute value of the reported distance should be interpreted as an implementation-dependent estimate rather than a calibrated metrological measurement.

From the perspective of device-free sensing, the key requirement is not absolute ranging accuracy but the stability of the baseline signal and the detectability of motion-induced perturbations. Variations in the multipath propagation environment caused by human movement introduce characteristic temporal deviations in the estimated distance signal that can be exploited as informative features for activity recognition and fall detection.

The sensing principle therefore relies on temporal perturbations of the estimated distance signal rather than on precise localization, as discussed in Section 3.

### 4.6. Data Acquisition and Labeling

BLE CS procedures were executed periodically at a fixed update rate. For each CS procedure, the Initiator logged the following information:

- a timestamp generated on the host computer,
- the estimated distance between the Initiator and Reflector obtained from phase-based ranging.

To enable reliable temporal alignment of walking, falling, and transitional events, all experimental scenarios were recorded in parallel using a video camera connected to a separate host PC. The BLE CS system and the video recording system operated independently; however, both host PCs were synchronized to the same network time server prior to data collection. Perfect fine-grained synchronization was not required, as individual activity events were well separated in time and segmented at a coarse temporal resolution. The video recordings were used exclusively for temporal segmentation and ground-truth labeling, and not as input to the proposed sensing or classification pipeline.

Three fall scenarios and their corresponding non-fall counterparts, as described in Section 4.4, were performed by six volunteer actors (three male and three female). All participants were healthy younger adults. Female participants had heights approximately in the range of 165–175 cm, and male participants approximately 175–185 cm. Volunteers were selected within typical adult height ranges and had normal body composition. Each scenario was repeated five times per actor, resulting in a total of 6×3×5=90 recorded trials. Each trial contains a continuous time series of distance estimates, with approximately 100 samples per trial, as reflected in the provided dataset.

Ground-truth labels were obtained through manual annotation of the video recordings. Based on the annotated timestamps, the corresponding CS data were segmented into overlapping temporal windows and labeled as either *fall* or *non-fall*, depending on the dominant activity within each window. Transitional periods surrounding loss-of-balance events were included to capture realistic dynamics rather than idealized motion patterns.

To evaluate subject-independent generalization, data from two male and two female actors were used for feature extraction and model training, while the remaining data from one male and one female actor were reserved exclusively for testing. This split ensures that the evaluation reflects performance on previously unseen individuals, which is critical for realistic deployment in assisted living environments.

This labeling strategy ensures reliable ground truth while preserving the device-free and privacy-aware nature of the proposed system.

The key acquisition and segmentation parameters are summarized in Table 1. These parameters define the temporal resolution of the CS measurements and the subsequent window-based feature extraction process.

The effective update rate of 10 Hz reflects the practical execution frequency of periodic BLE CS procedures under the selected configuration. This sampling rate provides sufficient temporal resolution to capture rapid fall dynamics while remaining compatible with low-power residential deployment constraints.

The window length of 2.0 s (20 samples) was selected to capture the full temporal structure of a fall event, which typically includes a short destabilization phase, rapid descent, and brief post-impact stabilization. Shorter windows risk truncating fall transients, whereas substantially longer windows would introduce unnecessary non-event motion.

A 50% overlap was adopted to improve temporal localization and reduce boundary effects, ensuring that fall transients occurring near window edges are fully represented in at least one segment. Although overlapping windows introduce temporal correlation, trial-level subject-independent splitting prevents leakage between training and test sets.

Trial durations varied between 8 and 12 s depending on the scenario and included pre-event motion, fall or recovery phase, and post-event stillness. This resulted in approximately 8–10 windows per trial under the selected segmentation parameters.

### 4.7. Feature Extraction and Classification

The raw output of the BLE CS system consists of a univariate time series of distance estimates between the Initiator and Reflector. Let

(1) $$d=\{d_{1},d_{2},\ldots ,d_{N}\}$$

denote a sequence of *N* distance samples within a temporal analysis window. Rather than operating directly on scalar distance estimates, a feature-based representation was adopted to capture characteristic temporal patterns associated with falls and non-fall activities while maintaining robustness to noise and minor environmental variations.

This feature-based approach follows established practice in device-free RF-based fall detection, where handcrafted time-domain features have been shown to effectively capture abrupt motion dynamics and post-event stillness, particularly in scenarios with limited training data and strong environmental variability [5,11,32].

Following ground-truth segmentation, the distance time series was divided into overlapping temporal windows. From each window, a set of statistical, temporal-variation, trend-related, and energy-based features was extracted, motivated by prior work in Wi-Fi CSI-, radar-, and RF-based human activity recognition [14,25].

#### 4.7.1. Statistical Features

Statistical descriptors characterize the overall dispersion and variability of distance estimates within a window, which tend to increase during falls due to rapid body displacement. The mean distance is defined as

(2) $$\mu_{d}=\frac{1}{N}\sum_{i=1}^{N}d_{i},$$

and the standard deviation is

(3) $$\sigma_{d}=\sqrt{\frac{1}{N-1}\sum_{i=1}^{N}{\left(d_{i}-\mu_{d}\right)}^{2}}.$$

The minimum and maximum distances are

(4) $$d_{\min}=\min_{i}d_{i},\;d_{\max}=\max_{i}d_{i},$$

with the signal range

(5) $$R_{d}=d_{\max}-d_{\min}.$$

To improve robustness against outliers, the interquartile range (IQR) is computed as

(6) $${\mathrm{IQR}}_{d}=Q_{75}\left(d\right)-Q_{25}\left(d\right),$$

where $Q_{25}$ and $Q_{75}$ denote the first and third quartiles, respectively. Similar statistical descriptors have been widely employed in RF-based fall detection systems to distinguish falls from controlled or supported movements [11,19].

#### 4.7.2. Temporal Variation Features

Falls are typically characterized by abrupt changes in body position caused by loss of balance and rapid descent. To capture such dynamics, the first-order difference sequence is computed as

(7) $$\Delta d_{i}=d_{i}-d_{i-1},\;i=2,\ldots ,N.$$

From this sequence, the mean absolute first difference is defined as

(8) $$\mu_{\Delta }=\frac{1}{N-1}\sum_{i=2}^{N}\left|\Delta d_{i}\right|,$$

and the maximum absolute first difference is

(9) $$\Delta_{\max}=\max_{i}\left|\Delta d_{i}\right|.$$

In addition, the variance of the first differences is computed as

(10) $$\sigma_{\Delta }^{2}=\frac{1}{N-2}\sum_{i=2}^{N}{\left(\Delta d_{i}-\bar{\Delta d}\right)}^{2},$$

where $\bar{\Delta d}$ denotes the mean of the first-difference sequence. Such temporal-derivative features explicitly target sudden motion transients and have been shown to be discriminative in Wi-Fi CSI- and radar-based fall detection [14,25].

#### 4.7.3. Trend-Related Features

After the initial impact, falls often exhibit sustained distance trends caused by sliding or collapsing motions followed by limited movement. To capture this behavior, a linear model is fitted to the windowed distance signal:

(11) $$d_{i}=a\;i+b+\varepsilon_{i},$$

where *a* is the slope, *b* is the intercept, and $\varepsilon_{i}$ is the residual error. The slope *a* reflects monotonic distance changes, while the mean squared residual error

(12) $$\mathrm{MSE}=\frac{1}{N}\sum_{i=1}^{N}\varepsilon_{i}^{2}$$

quantifies deviations from linearity. Similar trend-based descriptors have been used to distinguish falls from periodic or stationary activities in RF-based human motion sensing [25,32].

#### 4.7.4. Energy-Related Features

Energy-based measures summarize the overall magnitude of distance fluctuations within a window and provide a compact representation of motion intensity. The signal energy is defined as

(13) $$E_{d}=\sum_{i=1}^{N}d_{i}^{2},$$

and the root mean square (RMS) value is computed as

(14) $${\mathrm{RMS}}_{d}=\sqrt{\frac{1}{N}\sum_{i=1}^{N}d_{i}^{2}}.$$

Such features are commonly used in RF-based activity recognition to differentiate high-energy events such as falls from low-energy daily activities [11,19].

All extracted features were standardized using z-score normalization with statistics computed exclusively from the training data. This prevents information leakage from the test set and ensures consistent scaling across subjects and scenarios.

### 4.8. Classification Models

To evaluate the discriminative power and robustness of the extracted distance-based features, three supervised binary classifiers were considered, representing different model families commonly used in RF-based human activity and fall detection tasks: (i) a Support Vector Machine (SVM) with a radial basis function (RBF) kernel, (ii) a Random Forest (RF) classifier, and (iii) gradient-boosted decision trees implemented using XGBoost. All classifiers were trained and evaluated using identical window-level feature vectors and a subject-independent evaluation protocol.

#### 4.8.1. Support Vector Machine

Given the limited dataset size and the low-dimensional feature space, a Support Vector Machine with a radial basis function (RBF) kernel was employed, as SVMs have demonstrated strong generalization performance in RF-based fall detection and posture recognition tasks [33].

The RBF kernel is defined as

(15) $$K\left(x_{i},x_{j}\right)=\exp\;\left(-\gamma ∥x_{i}-x_{j}{∥}^{2}\right),$$

where γ controls the kernel width. Classification is performed by solving the soft-margin SVM optimization problem

(16) $$\min_{w,b,\xi }\;\frac{1}{2}{∥w∥}^{2}+C\sum_{i=1}^{N}\xi_{i},$$

subject to

(17) $$y_{i}\left(w^{⊤}\phi \left(x_{i}\right)+b\right)\geq 1-\xi_{i},\;\xi_{i}\geq 0,$$

where *C* is the regularization parameter controlling the trade-off between margin maximization and misclassification penalty.

#### 4.8.2. Random Forest

Random Forest classification was employed to capture nonlinear feature interactions without explicit kernel design. The ensemble consisted of multiple decision trees trained on bootstrap samples, with class-weight balancing applied to mitigate bias toward non-fall samples.

#### 4.8.3. XGBoost

Gradient-boosted decision trees were implemented using the XGBoost framework to model nonlinear relationships and feature interactions within the extracted distance-based feature space. XGBoost incrementally builds an ensemble of weak decision tree learners by optimizing a regularized objective function, enabling strong performance on structured, low-dimensional datasets and effective handling of class imbalance.

### 4.9. Cost-Sensitive Classification Strategy

Fall detection is an inherently asymmetric classification problem, in which different types of errors have unequal practical consequences. Missing a fall event (false negative) may lead to severe safety risks, whereas false alarms (false positives), although undesirable, are generally less critical. To reflect this asymmetry, a cost-sensitive, recall-oriented classification strategy was adopted.

During training, fall samples were assigned a higher class weight than non-fall samples, increasing the penalty associated with missed fall detections in the loss function. Let $N_{nf}$ and $N_{f}$ denote the number of non-fall and fall samples in the training dataset, respectively. The class weight for fall events was set proportional to the class imbalance ratio:

(18) $$w_{f}=\frac{N_{nf}}{N_{f}},$$

while the non-fall class weight was set to $w_{nf}=1$.

In addition to cost-sensitive training, the decision threshold applied to the classifier output probability was adjusted to further prioritize recall. A lower threshold than the conventional value of 0.5 was selected based on validation data, intentionally allowing a higher false positive rate in exchange for reduced missed detections. Model performance was therefore evaluated using recall-oriented metrics, including recall, precision–recall area under the curve (PR-AUC), and confusion matrices, rather than overall accuracy alone.

### 4.10. Evaluation Metrics

Fall detection systems deployed in assisted living environments must prioritize the reliable detection of true fall events, as missed detections may lead to severe medical consequences. Accordingly, system performance was evaluated using standard binary classification metrics derived from the confusion matrix, with particular emphasis on sensitivity (recall) for the *fall* class.

Let TP, TN, FP, and FN denote the number of true positives, true negatives, false positives, and false negatives, respectively. The following metrics were computed:

- Sensitivity (Recall):
  (19) $$\mathrm{Sensitivity}=\frac{\mathrm{TP}}{\mathrm{TP}+\mathrm{FN}},$$
  measuring the proportion of actual fall events correctly detected.
- Specificity:
  (20) $$\mathrm{Specificity}=\frac{\mathrm{TN}}{\mathrm{TN}+\mathrm{FP}},$$
  quantifying the system’s ability to correctly reject non-fall activities.
- Precision:
  (21) $$\mathrm{Precision}=\frac{\mathrm{TP}}{\mathrm{TP}+\mathrm{FP}},$$
  indicating the reliability of detected fall alarms.
- Accuracy:
  (22) $$\mathrm{Accuracy}=\frac{\mathrm{TP}+\mathrm{TN}}{\mathrm{TP}+\mathrm{TN}+\mathrm{FP}+\mathrm{FN}},$$
  providing an overall measure of classification correctness.
- F1-score:
  (23) $$F1=2\cdot \frac{\mathrm{Precision}\cdot \mathrm{Sensitivity}}{\mathrm{Precision}+\mathrm{Sensitivity}},$$
  balancing detection completeness and reliability.

While accuracy and F1-score are reported for completeness and comparability with prior work, sensitivity is treated as the primary performance indicator in this study, reflecting the application-driven requirement that failing to detect a fall is more critical than generating occasional false alarms [3,5].

### 4.11. Experimental Protocol

Model evaluation was conducted under a subject-independent protocol. Such protocol ensures that reported performance reflects generalization to previously unseen individuals rather than subject-specific motion patterns.

Classification was performed at the window level, followed by optional aggregation to the trial level using majority voting. This aggregation strategy reduces sensitivity to isolated misclassifications and better reflects event-level fall detection performance, which is more relevant for real-world deployment.

## 5. Experimental Results

This section presents the experimental evaluation of the proposed BLE CS–based fall detection system. Performance is assessed under a subject-independent protocol using the evaluation metrics defined in Section 4.8, with particular emphasis on fall detection sensitivity. Results are reported at the feature level, signal level, and classifier level to assess robustness across different movement patterns and learning models.

### 5.1. Distance Time Series Characteristics and Feature-Based Dataset Analysis

**Figure 2:**
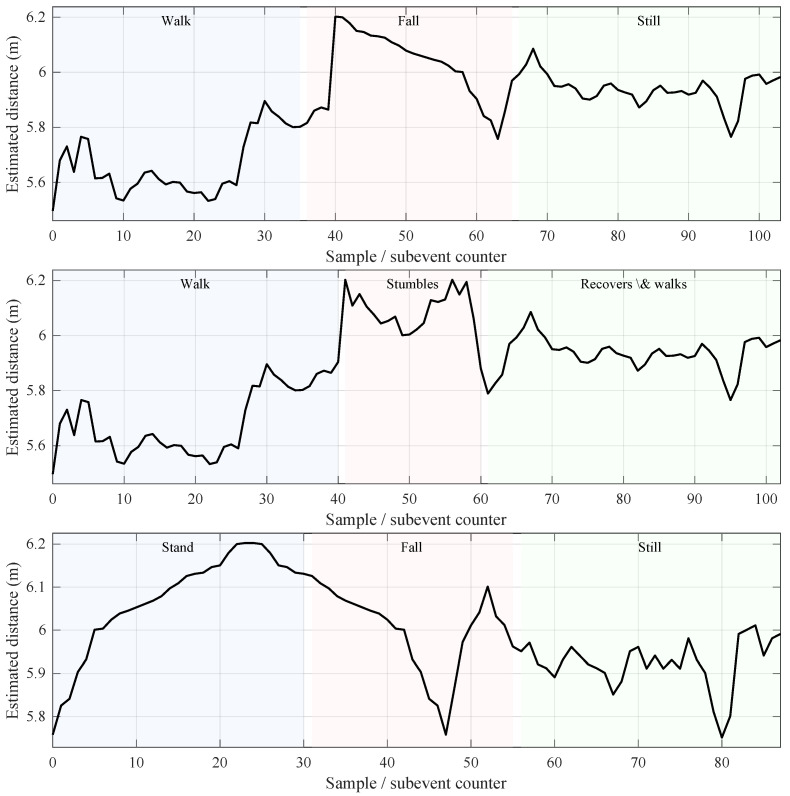
Representative BLE CS distance time series for selected fall and non-fall scenarios, illustrating characteristic temporal dynamics under realistic indoor multipath conditions.

To provide an intuitive understanding of the signals captured by BLE CS, Figure 2 shows representative examples of the recorded distance time series for selected fall and non-fall scenarios. The x-axis denotes the sample index within a trial, while the y-axis represents the estimated distance between the Initiator and Reflector. Despite the use of scalar distance estimates only, the signals exhibit distinct temporal patterns associated with different movement types, including abrupt distance changes during falls, smoother variations during supported walking, and relatively stable segments during post-event stillness.

Building on these observations, the dataset was quantitatively characterized using the feature set defined in Section 4. Table 2 reports mean feature values computed across trials for each scenario group. Even with distance-only sensing, the extracted features reveal systematic differences between scenarios, particularly in temporal variation metrics (e.g., $\mu_{\Delta }$ and $\Delta_{\max}$) and trend-related measures such as the mean squared error (MSE). These differences indicate that the proposed distance-based feature representation captures discriminative signal structure relevant for fall detection, providing a compact and interpretable basis for subsequent classification.

To further quantify the discriminative power of the extracted features, a relative separability indicator was computed for each feature *f* based on window-level statistics. Let $\mu_{f}^{\left(\mathrm{fall}\right)}$ and $\mu_{f}^{\left(\mathrm{non}\text{-}\mathrm{fall}\right)}$ denote the mean values of feature *f* over fall and non-fall windows, respectively. The separability is defined as:

(24) $$S_{f}=\frac{\left|\mu_{f}^{\left(\mathrm{fall}\right)}-\mu_{f}^{\left(\mathrm{non}\text{-}\mathrm{fall}\right)}\right|}{\sigma_{f}},$$

where $\sigma_{f}$ is the standard deviation of feature *f* computed over all windows. This metric provides a normalized, model-independent measure of class separation and allows direct comparison of the discriminative strength of different features.

**Figure 3:**
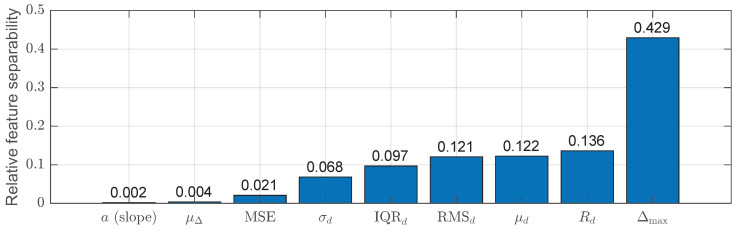
Relative separability of distance-based features between fall and non-fall windows. Separability is computed as the normalized absolute difference between the mean feature values of fall and non-fall classes, based on window-level statistics. The plot provides a model-agnostic indication of which time-domain features contribute most strongly to distinguishing fall events in the recorded dataset.

Figure 3 visualizes the relative separability of the extracted features, highlighting the contribution of temporal-variation and trend-related descriptors to fall discrimination. The separability measure shown in Figure 3 is computed from aggregated window-level feature statistics and is independent of any specific classifier. It is included to aid interpretability of the extracted features rather than to represent a learned feature importance score.

### 5.2. Classifier Comparison Under Sensitivity-First Evaluation

Given that missed fall detections may have severe consequences in elderly care, classifier evaluation was aligned with a sensitivity-first operating philosophy. In addition to the default operating point with a decision threshold of 0.5, a sensitivity-first operating mode was evaluated, in which the decision threshold is selected to satisfy a target recall (Sensitivity ≥0.95) on a validation set. All performance metrics are subsequently recomputed on a held-out test set using the selected threshold, ensuring that threshold selection does not bias test results.

Three supervised classifiers were compared using identical window-level feature vectors: (i) a Support Vector Machine (SVM) with a radial basis function (RBF) kernel, (ii) a Random Forest (RF) classifier, and (iii) gradient-boosted decision trees (XGBoost). These models represent commonly adopted baselines and state-of-the-art approaches for low-dimensional tabular learning in device-free sensing and fall detection, including WiFi CSI- and radar-based systems [11,14,19,25]. All models were trained exclusively on data from the training subjects, with feature normalization parameters computed using the training set only. Evaluation was performed on previously unseen subjects to assess subject-level generalization.

Hyperparameter optimization was performed exclusively within the training partition under the subject-independent split. Specifically, the training data (comprising only training subjects) were internally divided using cross-validation folds, and a grid search strategy was applied to select optimal hyperparameters for each classifier. For the SVM, the regularization parameter *C* and RBF kernel parameter γ were tuned. For the Random Forest, the number of trees, maximum tree depth, and minimum samples per split were explored. For XGBoost, the number of boosting rounds, learning rate, and maximum tree depth were optimized. Model selection was based on validation performance averaged across folds. The held-out test subjects were not used during hyperparameter selection, thereby preventing information leakage and ensuring unbiased performance evaluation.

### 5.3. Decision Thresholding

To explicitly evaluate a sensitivity-first operating regime, classifier decision thresholds were adjusted using a validation set to enforce a minimum target sensitivity. Let $\hat{p}\left(\mathrm{fall}|x\right)$ denote the predicted fall probability for a window-level feature vector *x*. In the default operating mode, binary decisions are obtained as

$$\hat{y}=I\left\{\hat{p}\left(\mathrm{fall}|x\right)\geq 0.5\right\}.$$

For sensitivity-first evaluation, the decision threshold τ is selected on the validation set such that

Sensitivity(τ)≥0.95.

Among all thresholds satisfying this constraint, the threshold maximizing specificity is selected. The chosen threshold τ is then fixed and applied to the independent test set.

The same cross-validation folds within the training partition were used for threshold selection. Thus, both hyperparameter optimization and decision threshold calibration were performed exclusively using data from training subjects. The held-out test subjects were not involved in any stage of model selection or threshold tuning, preventing information leakage between training and evaluation phases.

Table 3 reports window-level classification results obtained using the default decision threshold. At this operating point, all three classifiers achieve high sensitivity and specificity on the test set, indicating strong separability between fall and non-fall windows in the recorded dataset. XGBoost achieves the highest sensitivity and overall accuracy, while SVM and RF remain competitive.

It should be noted that the perfect specificity and precision values observed for certain classifiers at the default operating point reflect the limited size of the held-out test set and the controlled experimental conditions. With a finite number of non-fall windows, the absence of false positives in a given test split can result in 100% specificity or precision. Such results should therefore be interpreted cautiously and not as definitive evidence of real-world performance across larger and more diverse populations.

Table 4 summarizes performance under sensitivity-first operation. As expected, enforcing a high-recall constraint increases sensitivity to near-perfect or perfect levels for all classifiers, at the cost of reduced specificity and precision due to an increased number of false alarms. Such trade-offs are well documented in fall detection literature and are generally considered acceptable in assisted living scenarios, where missed detections pose greater risk than occasional false alarms [4,5]. Among the evaluated models, XGBoost provides the most favorable balance between sensitivity and specificity in this operating regime.

To place these results in quantitative context, the window-level performance can be compared with sensitivity ranges reported for device-free fall detection systems based on WiFi Channel State Information (CSI) and millimeter-wave radar sensing. The DeFall system [11], which explicitly addresses environment-independent WiFi-based fall detection, reports sensitivity values in the range of approximately 92–97% depending on the environment and evaluation protocol. The WiFall system [19] reports fall detection rates above 90% under controlled indoor conditions. A deep learning–based WiFi CSI approach [14] reports sensitivity values between 94–98%, although subsequent analysis has highlighted that performance may be overestimated when evaluation protocols do not strictly enforce cross-environment or subject-independent testing [20].

Millimeter-wave radar-based systems, such as the multi-view parameter fusion approach in [25], report sensitivity values typically between 92–99% depending on sensing configuration and signal representation. These systems leverage richer range–Doppler features and dedicated hardware to achieve strong motion discrimination.

In the experimental setting considered in this work, the proposed BLE CS approach achieved window-level sensitivity of 96.9% at the default operating point and 100.0% under sensitivity-first thresholding, with specificity between 90.6% and 100.0% depending on the selected operating regime. These values correspond to the dataset and single residential environment described in Section 4 and should therefore be interpreted as indicative results under the tested conditions rather than as universally representative performance.

To provide a consolidated quantitative overview, Table 5 summarizes representative sensitivity values reported in WiFi CSI- and radar-based systems alongside the results obtained in this study. Because the compared works use different environments, datasets, and evaluation protocols, the reported values should be interpreted as indicative performance ranges rather than directly comparable benchmark results.

## 6. Discussion

This work set out to investigate whether BLE CS, using only low-dimensional distance estimates and simple time-domain features, can support reliable and privacy-preserving fall detection in residential environments. When interpreted in the context of prior device-free fall detection studies, the results reported in Table 3 and Table 4 shed light on the feasibility and limitations of this sensing approach.

### 6.1. Dataset Size and Generalization Considerations

A common concern in data-driven fall detection research relates to the size and diversity of the experimental dataset used for training and evaluation. The dataset employed in this study comprises a limited number of actors and controlled residential environments, which reflects practical constraints associated with safely collecting realistic fall data. As noted in prior work, acquiring large-scale datasets involving genuine falls by elderly individuals is ethically, medically, and logistically challenging, and many existing studies therefore rely on simulated falls performed by young or healthy volunteers [5,18].

Rather than aiming to maximize dataset size, this work prioritizes ecological validity by focusing on realistic fall mechanisms and challenging non-fall activities performed in furnished, residential-like environments. The fall scenarios were designed to reflect common domestic situations, including wall-supported balance recovery, furniture-assisted locomotion, and sit-to-stand transitions, which are known to generate motion patterns that closely resemble true fall dynamics. This design choice is intended to reduce the risk of overly optimistic performance estimates that can arise when models are trained on simplified or exaggerated fall motions [20].

In addition, evaluation was conducted under a subject-independent protocol, ensuring that reported results reflect generalization to previously unseen individuals rather than subject-specific motion signatures. This evaluation strategy is widely regarded as more stringent and more representative of real-world deployment than subject-dependent testing, particularly in fall detection and human activity recognition tasks [11,14]. The use of relatively simple, low-dimensional feature representations and classical machine learning models further reduces the risk of overfitting compared to deep learning approaches trained on limited datasets.

It should be noted that overlapping windows introduce temporal correlation between adjacent segments within the same trial. To prevent data leakage, training and testing sets were separated at the subject level, ensuring that no windows derived from the same subject or trial appear in both partitions. Nevertheless, window-level evaluation may yield optimistic estimates compared to strict event-level assessment due to intra-trial dependency. The reported metrics should therefore be interpreted as window-level performance indicators under subject-independent validation.

It is important to emphasize that the objective of this work is not to claim state-of-the-art performance in absolute terms, but to demonstrate the feasibility of BLE CS as a device-free sensing modality for fall detection under realistic residential conditions. The reported results should therefore be interpreted as a proof-of-concept that establishes the discriminative potential of distance-based distance estimates derived from CS, rather than as a definitive assessment of performance in large-scale deployments.

Future work will focus on expanding the dataset across a broader range of environments, subject characteristics, and fall types, as well as investigating longitudinal data collection and cross-environment generalization. Such extensions are expected to further improve robustness and enable more comprehensive benchmarking against mature Wi-Fi- and radar-based fall detection systems.

### 6.2. Interpretation of Classification Results

Across all evaluated classifiers, high window-level sensitivity was achieved at the default operating point, with XGBoost achieving the strongest overall performance. These results support the underlying premise of this study: that fall-related motion dynamics induce characteristic temporal patterns in BLE CS distance estimates that are separable from non-fall activities using relatively simple features. Notably, this separability was achieved without relying on high-dimensional RF representations such as Wi-Fi CSI matrices or radar range–Doppler maps.

When shifting to a sensitivity-first operating regime, all classifiers were able to satisfy the target constraint of Sensitivity ≥0.95, at the expected cost of reduced specificity and precision. This behavior is consistent with prior fall detection studies, where relaxing decision thresholds to minimize missed detections inevitably increases false alarms [4,5]. From an assisted living perspective, this trade-off is often acceptable, as delayed or missed detection of a real fall poses significantly greater risk than occasional false alerts.

Although performance metrics are primarily reported at the window level, fall events occur at the trial level and typically span multiple overlapping windows. In this study, a fall event is considered detected if at least one window within the corresponding trial is classified as a fall. This conservative aggregation strategy is consistent with the sensitivity-first operating regime, where minimizing missed falls is prioritized over reducing false alarms. Alternative aggregation strategies, such as majority voting across windows or temporal smoothing, may further refine event-level specificity and represent an important direction for future investigation.

Among the evaluated models, XGBoost demonstrated the most favorable sensitivity–specificity balance under sensitivity-first operation. This aligns with observations in broader human activity recognition and RF sensing literature, where gradient-boosted trees often outperform classical classifiers on low-dimensional tabular feature spaces by effectively capturing nonlinear feature interactions [14]. Nevertheless, the strong performance of SVM and Random Forest classifiers indicates that the discriminative power arises primarily from the sensing modality and feature representation rather than from highly specialized model architectures.

It is important to note that perfect specificity observed at the default operating point reflects, in part, the limited dataset size and controlled experimental conditions. Similar effects have been reported in early-stage WiFi CSI and radar-based fall detection studies evaluated on small-scale or highly structured datasets [11,25]. As highlighted by recent analyses, performance reported under such conditions may overestimate real-world accuracy if not validated across diverse environments and activity patterns [20].

### 6.3. Comparison with Existing RF-Based Fall Detection Approaches

Table 6 provides a qualitative comparison between the proposed BLE CS approach and representative device-free fall detection systems based on WiFi CSI and millimeter-wave radar sensing.

WiFi CSI-based systems leverage fine-grained channel measurements that capture subtle human motion effects but typically require high-dimensional feature extraction, environment-specific calibration, and careful mitigation of performance overestimation [11,14]. Radar-based approaches offer strong sensitivity and robustness to lighting and occlusion but rely on dedicated hardware and more complex signal processing pipelines [25].

In contrast, the BLE CS-based approach explored in this work operates on a single scalar distance estimate per measurement, resulting in substantially lower data dimensionality and computational complexity. Despite this simplicity, the observed sensitivity-first performance falls within the range of sensitivity values reported for WiFi CSI- and radar-based systems in the literature. However, these systems have typically been validated on larger and more diverse datasets, and therefore direct one-to-one performance equivalence cannot be inferred from the present limited-scale evaluation.

Bluetooth Low Energy (BLE) development toolkits commonly provide RSSI-based proximity or coarse distance estimation APIs, typically relying on log-distance path loss models calibrated at short range. Such approaches are widely used for room-level localization and proximity detection. However, RSSI provides amplitude-only measurements that are highly sensitive to multipath fading, antenna orientation, and environmental variability, often exhibiting significant fluctuations even in static indoor conditions [21]. For this reason, modern device-free sensing systems for fall detection and fine-grained human activity recognition rely on richer channel representations such as WiFi CSI or radar range–Doppler features rather than RSSI [11,14,32]. In contrast to RSSI, BLE CS enables phase-based distance estimation and improved temporal stability, providing motion-relevant information that is fundamentally unavailable from amplitude-only metrics. Consequently, while RSSI-based proximity tools exist, their inherent signal limitations make them unsuitable as a competitive baseline for high-sensitivity device-free fall detection under realistic multipath conditions.

Several studies have investigated the use of Bluetooth signals for passive human sensing without requiring the monitored person to carry a device. For example, Iannizzotto et al. [34,35] demonstrated that variations in BLE received signal strength (RSSI) caused by human motion can be exploited for device-free presence detection and occupancy monitoring using machine learning techniques. Other works explored device-free human counting and identification using BLE signals [36,37]. These studies confirm that Bluetooth signals can support passive sensing applications in indoor environments.

However, most existing BLE-based device-free sensing approaches rely on RSSI measurements and focus on relatively simple sensing tasks such as presence detection, occupancy estimation, or human counting. RSSI provides only coarse channel information and is highly sensitive to environmental variability, which limits its suitability for more complex activity recognition tasks such as fall detection.

Bluetooth-based fall detection systems have also been proposed using other Bluetooth measurement modalities. For example, Wan et al. [38] proposed a fall detection system based on Bluetooth Angle-of-Arrival (AoA) measurements combined with machine learning classification of human postures. Their system reports fall detection accuracy of approximately 98%, but it requires the monitored person to wear a Bluetooth beacon device that continuously transmits signals.

Table 7 summarizes representative Bluetooth-based sensing approaches and highlights the differences between existing methods and the proposed system. In contrast to wearable AoA-based fall detection systems and RSSI-based passive sensing approaches, the method proposed in this work explores device-free fall detection using BLE CS measurements exchanged between two fixed infrastructure nodes.

These findings support the hypothesis that recent advances in BLE CS enable sufficient motion sensitivity for fall detection while offering advantages in deployment cost, energy consumption, and ease of installation.

### 6.4. Implications for Privacy-Preserving Home Monitoring

An important implication of these results lies in the balance between sensing capability and user acceptance. Vision-based systems continue to face significant privacy concerns despite strong performance [8], while wearable-based solutions suffer from compliance and long-term usability issues [6,18]. BLE CS offers a compelling alternative by providing device-free sensing with minimal information leakage, aligning well with privacy-aware smart home paradigms [10,13]. The low-complexity signal representation further facilitates on-device or edge-based processing, reducing the need for continuous data transmission.

### 6.5. Limitations and Future Research Directions

While the presented results are promising, several limitations must be acknowledged. First, the dataset size remains limited and the experiments were conducted within a single furnished residential-like living-room environment.

Although this setting incorporated typical domestic elements (e.g., walls, furniture, and multipath-inducing structures), it does not capture the full diversity of residential spaces such as bedrooms, kitchens, or bathrooms, which may differ in layout, material composition, and electromagnetic propagation characteristics. Consequently, the reported results should be interpreted as an initial feasibility assessment under representative but controlled indoor conditions. Future work will extend the evaluation to multiple room types and heterogeneous residential layouts in order to systematically assess cross-room generalization and robustness under varied multipath and environmental dynamics.

Indoor residential environments are inherently dynamic: furniture may be rearranged, doors and windows opened or closed, and additional occupants or electronic devices may intermittently alter the radio propagation conditions. Such changes can modify the multipath structure of the wireless channel and may affect the baseline characteristics of RF sensing signals. Because the proposed method relies primarily on temporal perturbations of the estimated distance signal rather than on absolute ranging values, moderate environmental changes are expected to have limited impact on fall-event detectability. Nevertheless, systematic evaluation under dynamically changing environmental conditions remains an important direction for future work.

Another practical consideration for BLE CS sensing systems is the influence of interference and calibration in the crowded 2.4 GHz ISM band. BLE CS shares this spectrum with several wireless technologies, including Wi-Fi access points, Bluetooth devices, and other short-range communication systems. Consequently, channel sounding measurements may be affected by external interference and dynamic spectrum occupancy. Experimental studies have shown that the impact of interference depends on factors such as signal bandwidth, power level, and temporal characteristics. For example, Sheikh [31] demonstrates that strong or wideband interference in the ISM band can affect the stability of BLE CS ranging measurements.

Calibration and hardware-specific factors may also influence the absolute accuracy of estimated distances. Device-dependent phase offsets, antenna characteristics, and synchronization imperfections can introduce systematic bias in the reported distance estimates. Experimental evaluation on commercial hardware by Wieme et al. [30] highlights the importance of calibration and implementation details for accurate ranging. Similar observations regarding environmental sensitivity and implementation-dependent behavior of channel sounding techniques have been reported in several recent studies [26,27,28,39,40].

In the present study, the experiments were intentionally conducted in a realistic indoor environment rather than in an RF-isolated laboratory setup. The measurement room was located within a university building where multiple wireless systems were operating in nearby rooms and corridors. Furthermore, the fall detection experiments were performed across several experimental sessions over multiple days, and the sensing devices were repositioned between sessions. This resulted in small variations in the baseline estimated distance between the devices.

Despite these variations in interference conditions and device placement, the short-term variability of the estimated distance signal under static conditions remained comparable across sessions, while fall events consistently produced transient deviations substantially exceeding the baseline variability. These observations suggest that moderate interference and calibration offsets primarily influence the absolute distance estimate, whereas the temporal perturbations of the signal caused by human motion remain detectable and informative for fall detection.

Future work should include larger-scale data collection across multiple homes, room layouts, and occupant profiles to assess robustness and generalization. Second, ground truth labeling was available at the trial or segment level; finer-grained annotations could enable more precise modeling of near-fall events and recovery dynamics.

Future research directions include exploring multimodal fusion of BLE CS with complementary low-cost sensors (e.g., inertial beacons or ambient RF measurements), investigating adaptive thresholding strategies based on contextual information, and extending the approach to continuous long-term monitoring scenarios. Additionally, comparative studies using identical datasets across BLE, WiFi, and radar modalities would further clarify the relative strengths and limitations of each sensing approach under realistic deployment conditions.

## 7. Conclusions

This paper investigated the feasibility of BLE version 6.0 CS as a device-free and privacy-preserving sensing modality for fall detection in residential environments. Motivated by recent advances in standardized phase- and time-based Bluetooth measurements, we proposed a fall detection framework based on distance estimates derived from CS, combined with lightweight time-domain feature extraction and classical machine learning classifiers. Experimental results obtained under a subject-independent evaluation protocol demonstrate that BLE CS captures discriminative motion signatures associated with realistic fall events, achieving high sensitivity under the evaluated conditions while maintaining low deployment complexity and energy consumption.

In contrast to vision- and wearable-based approaches, the proposed system avoids cameras, microphones, and on-body devices, making it inherently compatible with privacy-by-design principles and suitable for long-term in-home monitoring. Compared to Wi-Fi CSI and radar-based sensing, BLE CS offers a favorable trade-off between sensing fidelity and practical deployability, leveraging ubiquitous low-power hardware and standardized protocols.

The system was deliberately optimized for a sensitivity-first operating regime, prioritizing the reliable detection of fall events over strict false alarm suppression. While this design choice may lead to an increased number of false positives in certain scenarios, such trade-offs are generally acceptable in assisted living contexts, where missed fall detections pose substantially higher risk than occasional false alarms.

The experimental evaluation was conducted on a limited-scale dataset comprising a small number of healthy adult participants recorded within a single residential-like environment. Consequently, the reported performance should be interpreted as a proof-of-concept demonstration of feasibility under controlled conditions rather than as definitive evidence of robustness across diverse real-world settings.

Achieving reliable large-scale deployment will require evaluation across broader subject populations, multiple residential layouts, varying environmental dynamics, and longitudinal data collection. In addition, future work should include systematic cross-environment validation and event-level performance assessment to further quantify generalization capability under realistic operating conditions.

Future work will focus on expanding the dataset across a broader range of environments and subject characteristics, investigating cross-environment generalization, and exploring the use of richer CS information beyond scalar distance estimates. In addition, integrating BLE CS with complementary low-cost sensing modalities and studying long-term, real-world deployments will be important steps toward practical adoption in assisted living applications.

## Figures and Tables

**Table 1:** Summary of data acquisition and windowing parameters used in the experiments.

Parameter	Value
Effective CS update rate	10 Hz
Window length	20 samples (2.0 s)
Window overlap	50% (1.0 s step size)
Average trial duration	9.8 s (range: 8–12 s)
Average windows per trial	8–10
Training/Test split	4 subjects (train), 2 subjects (test)

**Table 2:** Mean feature values per scenario group computed from the recorded distance time series.

	$\mu_{d}$	$\sigma_{d}$	$R_{d}$	IQR_*d*_	*μ*_Δ_	Δ_max_	*a* (Slope)	MSE	RMS_*d*_
Walk–Fall–Still	5.865	0.182	0.791	0.231	0.051	0.387	0.0036	0.0210	5.868
Walk–Stumble–Still	5.864	0.183	0.710	0.244	0.045	0.304	0.0038	0.0201	5.867
Stand–Fall–Still	5.995	0.110	0.488	0.154	0.036	0.156	−0.0019	0.0096	5.996
Stand–Walk–Still	5.863	0.183	0.717	0.246	0.046	0.705	0.0002	0.0330	5.866

**Table 3:** Window-level classification results using the default decision threshold (0.5).

Model	Sensitivity	Specificity	Precision	F1-Score	Accuracy
SVM (RBF)	90.6%	100.0%	100.0%	95.1%	95.3%
RF	93.8%	100.0%	100.0%	96.8%	96.9%
XGBoost	96.9%	100.0%	100.0%	98.4%	98.4%

**Table 4:** Window-level classification results under sensitivity-first evaluation (threshold selected to ensure Sensitivity ≥0.95 on validation data).

Model	τ	Sensitivity	Specificity	Precision	F1-Score	Accuracy
SVM (RBF)	0.38	96.9%	92.2%	93.9%	95.4%	94.5%
RF	0.31	100.0%	90.6%	91.4%	95.5%	95.3%
XGBoost	0.27	100.0%	93.8%	94.1%	97.0%	96.9%

**Table 5:** Quantitative comparison of representative device-free fall detection approaches reported in the literature. Reported sensitivity values correspond to fall-event recall as described in the respective studies and depend on the experimental environment, dataset size, and evaluation protocol used in each work.

Method	Modality	Reported Sensitivity	Reference
DeFall	WiFi CSI	92–97%	[11]
WiFall	WiFi CSI	>90%	[19]
DL-based CSI	WiFi CSI	94–98%	[14]
CSI performance analysis	WiFi CSI	Overestimation discussed	[20]
Multi-view mmWave radar	Radar	92–99%	[25]
This work (default)	BLE Ch. Snd.	96.9%	–
This work (sens.-first)	BLE Ch. Snd.	100.0%	–

**Table 6:** Qualitative comparison of device-free RF-based fall detection approaches.

Aspect	WiFi CSI	mmWave Radar	BLE Ch. Snd.
Dedicated sensing hardware	No	Yes	No
Signal dimensionality	High (CSI matrices)	High (range–Doppler)	Low (scalar distance)
Installation complexity	Medium	High	Low
Privacy preservation	High	High	High
Computational complexity	High	Medium– High	Low
Typical sensitivity (reported)	90–98%	92–99%	95–100% (this work)
Representative studies	[11,14,19]	[25]	This work

**Table 7:** Comparison of representative Bluetooth-based human sensing approaches.

Method	Technology	Device-Free	Application	Reported Accuracy
[34]	BLE RSSI perturbations	Yes	Presence detection/passive sensing	∼92–96%
[35]	BLE RSSI + deep learning	Yes	Occupancy detection	∼94–97%
[36]	BLE RSSI variations	Yes	Human counting	∼90%
[38]	Bluetooth AoA direction finding	No (wearable beacon required)	Fall detection and posture classification	98.14%
This work	BLE CS phase-based ranging	Yes	Device-free fall detection	96.9% accuracy, 98.4% F1-score

## Data Availability

The measurement dataset generated during the current study involves human participants and is available in anonymized form from the corresponding author upon reasonable request. Although no personal or identifiable data were recorded, controlled access is maintained to ensure compliance with institutional research governance policies. Core processing steps are fully described in the manuscript. Additional implementation details can be provided upon reasonable request.

## Abbreviations

The following abbreviations are used in this manuscript:

AAL	Ambient Assisted Living
BLE	Bluetooth Low Energy
CS	Channel Sounding
CSI	Channel State Information (Wi-Fi)
GDPR	General Data Protection Regulation
RF	Radio Frequency
RSSI	Received Signal Strength Indicator
SVM	Support Vector Machine
UWB	Ultra-Wideband
